# ‘I didn’t know what to expect’: Exploring patient perspectives to identify targets for change to improve telephone-delivered psychological interventions

**DOI:** 10.1186/s12888-020-02564-6

**Published:** 2020-04-07

**Authors:** Kelly Rushton, Kerry Ardern, Elinor Hopkin, Charlotte Welsh, Judith Gellatly, Cintia Faija, Christopher J. Armitage, Nicky Lidbetter, Karina Lovell, Peter Bower, Penny Bee

**Affiliations:** 1 School of Health Sciences, Division of Nursing, Midwifery and Social Work, Manchester Academic Health Science Centre, University of Manchester, Manchester, UK; 2 Department of Psychology, University of Sheffield, Sheffield, UK; 3 Manchester Centre for Health Psychology, School of Health Sciences, University of Manchester, United Kingdom; Manchester University NHS Foundation Trust, Manchester Academic Health Science Centre, Manchester, UK; 4 Anxiety UK, Manchester, UK; 5 NIHR School for Primary Care Research, Centre for Primary Care and Health Services Research, Manchester Academic Health Science Centre, University of Manchester, Manchester, UK

**Keywords:** Telephone therapy, Anxiety, Depression, Psychological wellbeing practitioner, IAPT, Mental health, Patient perspective, Common mental health problems, Guided self-help

## Abstract

**Background:**

Remote delivery of psychological interventions to meet growing demand has been increasing worldwide. Telephone-delivered psychological treatment has been shown to be equally effective and as satisfactory to patients as face-to-face treatment. Despite robust research evidence, however, obstacles remain to the acceptance of telephone-delivered treatment in practice. This study aimed to explore those issues using a phenomenological approach from a patient perspective to identify areas for change in current provision through the use of theoretically based acceptability and behaviour change frameworks.

**Methods:**

Twenty-eight semi-structured interviews with patients experiencing symptoms of common mental health problems, waiting, receiving or having recently received telephone-delivered psychological treatment via the UK National Health Service’s Improving Access to Psychological Therapies (IAPT) programme. Interviews were recorded, transcribed verbatim, and analysed using the Theoretical Domains Framework (TDF) and Theoretical Framework of Acceptability (TFA).

**Results:**

The majority of data clustered within five key domains of the TDF (knowledge, skills, cognitive and interpersonal, environmental context and resources, beliefs about capabilities, beliefs about consequences) and mapped to all constructs of the TFA (affective attitude, ethicality, intervention coherence, self-efficacy, burden, opportunity costs, and perceived effectiveness). Themes highlighted that early stages of treatment can be affected by lack of patient knowledge and understanding, reservations about treatment efficacy, and practical obstacles such as absent non-verbal communication, which is deemed important in the development of therapeutic alliance. Yet post-treatment, patients can reflect more positively, and report gaining benefit from treatment. However, despite this, many patients say that if they were to return for future treatment, they would choose to see a practitioner face-to-face.

**Conclusions:**

Using a combination of theoretically underpinned models has allowed the identification of key targets for change. Addressing knowledge deficits to shift attitudes, highlighting the merits of telephone delivered treatment and addressing skills and practical issues may increase acceptability of, and engagement with, telephone-delivered treatment.

## Background

Depression and anxiety are highly prevalent, and are associated with a substantial personal, economic and social burden [1]. Whilst there is a robust evidence base for psychological therapies, access is problematic due to a shortage of resources, inequalities in their distribution and inefficiencies in the utilisation of available means [2, 3]. To maximise the availability, accessibility and cost-effectiveness of mental health services whilst remaining clinically effective, it has been necessary to innovate in treatment delivery [4].

With the availability of technology, remote health care (such as treatment over the telephone) improves access for people who struggle to attend face-to-face appointments due to health, financial, time and situational difficulties [5–9] and can increase flexibility, accessibility and perceived anonymity (not being seen in person) [10–12].

A recent systematic review and meta-analysis of telephone delivered psychotherapy for depression highlighted adequate treatment adherence, beneficial effects when compared with usual care, and comparative effectiveness to face-to-face treatment [13]. In addition, patients report high levels of acceptance of telephone delivered psychological interventions [11] and when compared with the same treatment face-to-face, report equal satisfaction [14, 15].

Despite the research evidence, individual and societal attitudes influence both practitioners’ and patients’ acceptance of remote health care. Views rooted in the stereotypical traditional model of psychological treatment (i.e. face-to-face with a therapist) are largely influenced by mass media as the predominant source of information for many people [16, 17] which may impact upon the acceptance of alternative delivery methods of treatment. Existing literature on patients’ perspectives on remote health care has explored sociodemographic and clinical factors with less attention paid to individual viewpoints or system influences (human-technology, organisational, social, task and environmental factors [18]). To improve the design and implementation of remote health care, gaining an understanding of the roles individual and communal attitudes may play in uptake and use is essential.

In the United Kingdom (UK), the flagship Improving Access to Psychological Therapies (IAPT) programme has substantially increased access to evidence-based psychological therapies [19] using a stepped-care model. Low intensity (step 2) interventions (i.e. guided self-help) are delivered through a variety of modalities (face-to-face, telephone, groups, online), and large scale IAPT data sets show comparable outcomes between completed face-to-face and telephone treatments [20]. Due to the increasing demand on IAPT services in the UK, the use of telephone delivered interventions at step 2 is increasing. Yet despite a strong evidence base and comparable outcomes, there has been variable uptake in telephone interventions both nationally and internationally [21].

Quality of care can be defined by treatment effectiveness, clinical safety, and patient experience [22]. While the evidence for efficacy and safety of telephone-delivered treatment is accepted, in order to improve quality it is essential to understand the barriers and facilitators to engagement from a patient perspective. It has been increasingly acknowledged that consideration of patient acceptability using theories of behaviour change are key to effective evaluation, design and implementation of healthcare interventions [23–25] . The present study is the first to explore patient perspectives of telephone-delivered treatments from a combined acceptability and behaviour change lens.

Elements of the Theoretical Domains Framework (TDF [24]) and the Theoretical Framework of Acceptability (TFA [23]) will be used to provide a unique, theory-based account of the essential targets for a behaviour change intervention. The TDF draws together 33 behaviour change theories and 128 theoretical constructs to provide a robust framework to target behaviour change. The TFA provides an objective, evidence-based assessment and evaluation of the acceptability of a given intervention by considering seven aspects of acceptability synthesised from the literature exploring acceptability of the implementation of healthcare interventions Combining these two perspectives will provide an understanding of (i) which areas to target for change and (ii) the components within those areas that currently have low patient acceptability. This approach will enable the development of an intervention targeting the issues with the greatest capacity to improve acceptability, which in turn may improve engagement with telephone treatment.

## Methods

### Study design

This qualitative study is part of a wider research programme designed to enhance the quality of psychological interventions delivered by telephone (EQUITy). Semi-structured interviews were used to explore the views of patients who had been offered or received psychological treatment for common mental health problems (e.g. anxiety, depression) over the telephone in the last 12 months. The study took a phenomenological approach in order to capture participant’s individual unique experiences and perceptions of telephone treatment. Methods and results are presented in line with the Consolidated Guidelines for the Reporting of Qualitative Data [26]. The study was approved by the North West – Greater Manchester West Research Ethics Committee (Ref: 18/NW/0372).

### Recruitment

Participants were recruited via participating IAPT services and advertisement by third sector organisations, University bulletins and social media. To be eligible to take part, participants must have been offered or have received telephone-delivered psychological treatment within the previous 12 months. In order to achieve a sample representative of the general IAPT population, there were no other eligibility criteria for inclusion into the study. Recruitment through IAPT services involved distribution of information packs containing a consent-to-contact form to patients currently receiving or recently finished treatment. Following return of consent-to-contact forms to the research team, consent and demographic forms were sent and returned by email or post, in advance of interview. Recruitment via social media, bulletins and third sector organisations followed the same procedure after receiving an expression of interest from potential participants. Convenience sampling was conducted through the issuance of 500 invitations across 7 IAPT services; 32 people expressed interest in the study, of whom 28 went on to provide consent (see Table 1 for participant details) Within the sample, participant’s had varying amounts of previous psychological treatment experience face-to-face, online and through support calls via their employer, but none had accessed telephone treatment in IAPT prior to their current/recent treatment. Participants’ also passively disclosed information relating to medication they may have been prescribed for mental health issues, but this was not explored in the current study. Reasons for non-participation in the study included non-response following return of a consent to contact form (3) and a decision to withdraw for personal reasons (1). Additional recruitment was halted once data saturation was achieved during analysis when new themes ceased to emerge.

**Table 1:** Participant details

		Number of patients
Gender	Male	5
	Female	23
Age	18–29	8
	30–39	10
	40–49	2
	50–59	6
	60–69	2
Ethnicity	White British	23
	Asian British	2
	Other	3
Conditions^a^	Anxiety	8
	Depression	3
	Mixed anxiety/depression	6
	Other	2
	Don’t know	4
	No answer provided	5
Reason given for receiving telephone treatment	Shorter waiting list than face-to-face	8
	To avoid attending group therapy	4
	No alternative choice offered to patient	7
	Perceived anonymity	4
	Convenience	3
	Condition (anxiety/obsessive compulsive disorder)	2
Stage of treatment	Waiting for treatment	3
	Currently in treatment	4
	Completed treatment^b^	21

^a^Participants’ conditions were self-reported and not formal diagnoses, collected via a demographic pro forma

^b^Participants had completed at least two telephone sessions

### Procedure

Interviews were carried out between September 2018 and January 2019 by two female researchers, KR and KA. No prior relationship existed between the researchers and interviewees. Participants were offered an interview at a time convenient for them; the method employed for interview (face-to-face or telephone) was dependent on participant preference and distance from the study base. Four of the interviews were conducted face-to-face, 24 by telephone. Interviews lasted between 20 and 47 min and were digitally recorded, then transcribed verbatim by a University approved transcribing service. Participants had the opportunity to ask the research team questions prior to consent.

Interviews followed a semi-structured schedule, taking into consideration elements of the TDF [24], and TFA [23]. Once drafted, interview schedules were reviewed by the EQUITy Lived Experience Advisory Panel (LEAP). See Additional File 1 for the interview schedule. Interviews were focussed around participants’ initial perceptions of being offered telephone-delivered treatment, their beliefs, knowledge and understanding of what would happen, and their reflections on their experience of treatment.

### Analysis

The analysis was undertaken by an experienced team of researchers. KR (PhD) is a Mental Health Researcher, KA (MSc) is a Psychology Research Assistant and Psychological Wellbeing Practitioner (PWP), JG (PhD) is a Mental Health Researcher and Programme Manager, EH (BA) is a Health Research Administrator, CW (MSc) is a Service User Researcher and CF (PhD) is a Mental Health Researcher. All authors remained conscious of their position in relation to the research and worked to remain impartial during the collection, analysis and interpretation of data.

Data from interviews were analysed in a two-stage process, facilitated by NVivo software [27]. Initially, anonymised transcripts were analysed thematically using Braun and Clarke’s six-step process [28]. Transcripts were divided between four researchers KR, KA, EH and CW who first familiarised themselves with the data by reading and re-reading the transcripts. Each transcript was then coded inductively, and checked by two of the four researchers independently to generate an initial list of codes. Researchers met regularly as a group to merge coding files, remove duplicate codes and identify commonalities and relationships between codes to begin developing preliminary themes. Once initial coding was complete, KR, KA, EH and JG met to refine themes to ensure accurate reflection of participant data. Any discrepancies were discussed as a group to reach agreement. The completed thematic framework was finalised by KR for the second stage analysis.

Following thematic analysis, themes and constituent codes were mapped to the TDF [24] by KR, EH and KA. Mapping was undertaken by determining the ‘fit’ of themes and codes with domains using domain descriptions as working definitions [25].Once mapping was complete, data were checked a final time by KR and then validated by JG to confirm fit with the framework; any discrepancies during the mapping process were resolved through discussion. No identified themes fell outside of the TDF. Finally, themes within TDF domains were cross-mapped to the TFA by KR to identify areas of overlap and conflict. Themes were mapped to the TFA using the same method of ‘fit’ with TFA construct descriptions as working definitions [23]. Subthemes remained the same between the TDF and TFA, and once mapped were then discussed and agreed between the wider research team. No domains were excluded a priori and all domains of the TDF and the TFA were endorsed by the data to greater and lesser extents.

## Results

**Fig. 1:**
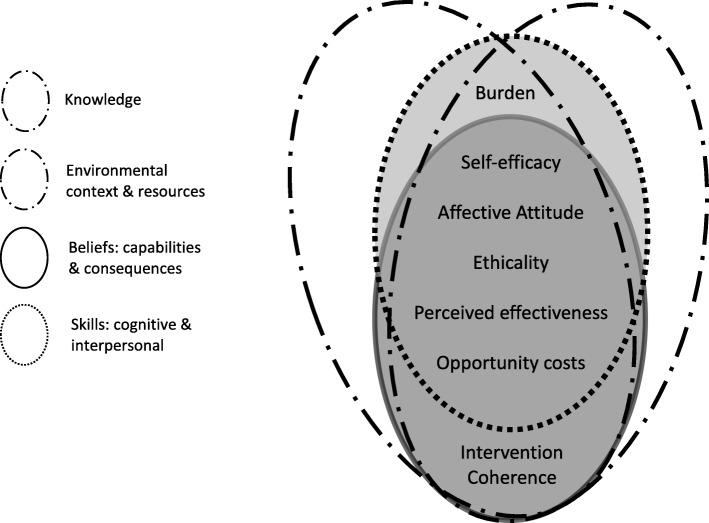
The interdependent relationship between key domains of the TDF ([24] in which data were clustered (ovals), and the related TFA [23] constructs (centre text)

A blended interpretation of the data is provided here to illustrate major areas to target for behaviour change in terms of patient acceptability (Fig. 1). Data broadly clustered across 5 of the 14 domains of the TDF and the 7 constructs of the TFA (Table 2). Four key target areas for change were identified following cross mapping of the two frameworks; Patient knowledge and understanding (TDF: Knowledge), patient and practitioner skills (TDF: Skills, Cognitive & Interpersonal), practical and environmental factors (TDF: Environmental Context and Resources), and beliefs (TDF: Beliefs about Capabilities, Beliefs about Consequences). Full details of data mapped to the TDF and TFA are presented in additional file 1.

**Table 2:** Key domains of the TDF in which data clustered, and related TFA constructs. Data were mapped within and between domains and constructs of the TDF/TFA, with bi-directional interactions. Descriptions are adapted from the original descriptions [23, 24]

**TDF Domain**	**Description**
Knowledge	An awareness and/or understanding of telephone treatment/practitioner.
Skills, cognitive & interpersonal	An ability or proficiency for taking part in telephone treatment sessions/practitioner and patient skills.
Environmental context & resources	Any circumstances related to the medium of telephone for treatment that affects the ability to successfully take part in a session.
Beliefs about capabilities/ consequences	Acceptance of the truth, reality or validity about an ability or facility to successfully partake or benefit from telephone treatment/about outcomes of partaking/the benefit of telephone treatment
**TFA Construct**	**Description**
Affective attitude	How an individual feels about telephone treatment
Burden	The perceived amount of effort that is required to participate in telephone treatment
Ethicality	The extent to which the telephone treatment has good fit with an individual’s value system
Intervention coherence	The extent to which the patient understands the telephone treatment and how it works
Opportunity costs	The extent to which benefits, profits or values must be given up to engage in the telephone treatment
Perceived effectiveness	The extent to which the telephone treatment is perceived as likely to achieve its purpose
Self-efficacy	The patient’s confidence that they can perform the behaviour(s) required to participate in telephone treatment

### Patient knowledge and understanding - lack of knowledge forms the foundation for preconceptions, inaccurate beliefs and anxiety

Considerable lack of knowledge around services, treatment options, practitioners and procedures underpinned many core issues, and significantly influenced the level of prospective acceptability of telephone treatment. Data relating to the TDF domain knowledge was linked with aspects of acceptability across all seven constructs of the TFA, and hinged predominantly around intervention coherence.

At the point of referral, patients’ understanding of treatment was markedly different to what was available (TFA: Intervention Coherence). Discourse describing general practitioner (GP) suggestions of ‘*going to talk to someone*’ contributed to pre-existing expectations of receiving face-to-face therapy. Phrases such as ‘*needing a listening ear*’, needing to ‘*offload*’, and stereotypes such as ‘*lying on a couch in a room somewher*e’ illustrated preconceptions about psychological treatment, which were not representative of treatment delivered at step 2. An overall lack of knowledge regarding the active participation required in treatment was apparent across patients both with and without prior experience of psychological treatment (TFA: Burden):

*I expected the therapy to sort of be let’s say about an hour a session, and I imagined it would be quite a lot of talking would be done, whereas I felt it was very much, well, I’ve emailed you the literature, now get on and read it.*

*(Participant 12, completed treatment)*

Lack of knowledge of step 2 treatment, particularly to delivery of that treatment by telephone up until the point of assessment appeared to play a role in the level to which it was deemed acceptable (TFA: Affective Attitude, Ethicality). Descriptions of feeling ‘pushed’ into telephone treatment, or its promotion via the incentive of shorter waiting lists were common reasons for embarking on telephone treatment. Despite a proportion of patients actively choosing telephone treatment for flexibility and convenience, feelings of desperation and the necessity for immediate help were significant drivers, based on knowledge of quicker access to treatment (TFA: Opportunity Costs). The sense of accepting something reluctantly chosen, based on a variable intimating lack of popularity, (i.e. shorter waiting lists than face-to-face) contributed to the perception of telephone treatment as inferior and often led to anxiety (TFA: perceived effectiveness, self-efficacy, affective attitude).

*‘It was more in making my decision, should I opt for face-to-face or should I go for telephone? That was where I had slight reservations about, oh, if I go for telephone will I get as much out of it or is it better to wait the longer waiting time and then get a face-to-face and it possibly will be more effective?’*

*(Participant 18, completed treatment)*

A lack of knowledge and understanding about treatment over the telephone, and previous experience of psychological treatment (e.g. counselling, cognitive behavioural therapy) appeared to underpin many of the reservations held by participants, who talked about feeling uncertain as to whether they would just speak and be listened to, or whether they would be told what to do to feel better (TFA: Intervention coherence). However, one participant spoke positively about expectations for telephone treatment, based on information provided at assessment, illustrating the relationship between knowledge and acceptability. Discourse centred on the *‘impression it was a format that worked’* highlighting that patient awareness of new treatments can significantly influence shifts in attitude towards increased acceptance (TFA: Intervention Coherence, Affective Attitude, Perceived Effectiveness), especially where those treatments become embedded in routine treatment provision*; ‘they [practitioners] were used to it’.*

Suspiciousness and scepticism stemming from deficits in knowledge appeared to contribute towards the development of inaccurate beliefs held by patients. The assumption that practitioners working on the telephone would be less qualified than those working face-to-face alluded to the pervasive perception that face-to-face is a superior treatment option (TFA: ethicality). The notion that more highly qualified practitioners would not be satisfied working on the telephone, and the idea that face-to-face treatment requires more advanced skills, was a commonly held belief:

*‘I think I always had that in the back of my head that I wasn’t sure that the person I was speaking to would have been considered qualified enough to do face-to-face counselling…I would hope that the people doing face-to-face stuff have more experience and have more training in psychological interventions…but I am assuming that someone doing psychological interventions over the phone will have had some training but they won’t be anywhere near as qualified or as experienced as someone who should be doing face-to-face interventions.’*

*(Participant 6, completed treatment)*

However, there were a number of participants who did believe that they would be talking to a qualified practitioner, and felt unconcerned by their initial lack of awareness of treatment, although those participants had often chosen telephone treatment for accessibility (TFA: opportunity costs). In this group, reduced prospective acceptability due to lack of knowledge was counterbalanced by the reduced perceived burden and opportunity cost afforded by telephone treatment.

Some knowledge of the practitioner delivering treatment was one significant factor for participants felt to be lost through telephone treatment. Awareness of a practitioner’s qualifications or even simple information such as knowing their face were considered important, by some, for initial engagement. In line with previous findings [11], in some situations people felt that seeing that a practitioner appeared professional, within a professional environment, would provide enough reassurance that they were sufficiently qualified, regardless of whether they were presented with the evidence of qualification (TFA: Ethicality):

‘*I didn’t really know who I was going to be speaking to. Because I’ve never done it before, I’ve never picked up the phone and spoken to somebody that I don’t know about this topic, that kind of freaked me out a bit. Especially if I don’t know who’s on the other end, I don’t know if they really are trained. I don’t know if they really have the qualifications that they say they have because I can’t see them.’*

*(Participant 24, partially completed treatment)*

Use of the word ‘stranger’ for describing practitioners on the telephone reflected the sense that it was unusual to open up about things that were often difficult to talk about, with someone that was *‘just a voice’,* who they had never met, and knew nothing about (TFA: Affective Attitude). Suggestions to alleviate such anxieties included meeting practitioners in person for first sessions, which was anticipated to facilitate development of a therapeutic relationship, or to offer patients a photograph and some background information about their practitioner, before commencing treatment. The disadvantage of practitioner anonymity in telephone treatment contrasted with the perceived benefits of telephone treatment in providing a sense of patient anonymity. While some participants spoke about the importance of needing to ‘know’ their practitioner, the potential to avoid felt stigma or conflict (i.e. family disapproval of treatment) through the perceived anonymity of telephone treatment, were highlighted as important advantages. Similarly, one participant felt that a distinct advantage of telephone treatment was that he had no idea who his practitioner was which enabled him to ‘open up’, and another highlighted the benefit of telephone treatment in preventing the tendency to make a judgement based on appearance. Such varying opinions demonstrate the challenges faced in identifying areas for change, and indicate the importance of a move towards more individualised care to improve acceptability.

For numerous participants, a general absence of understanding and awareness of telephone treatment appeared to have a significant influence over the level of anxiety felt about both commencing treatment and how effective it might be (TFA: Affective Attitude, Perceived Effectiveness). Preconceptions and assumptions about treatment appeared to be rooted in information gleaned from popular media, reinforced by language used at point of referral by primary care practitioners. Additionally, the role of the telephone in daily life (such as customer service call centres) contributed to the perception of telephone treatment being a poor relation to face-to-face provision, and a type of working that ‘properly qualified’ practitioners would not do.

‘*I felt like if I was going to a psychologist, going to a therapist physically, it would be a better psychologist because it would just be someone more professional. Because you know the assumption is if someone’s giving you this therapy over the phone, compared to someone in a professional office giving it to you face-to-face – like you see in movies and stuff like that, for example – it just seems the cheaper option. So you just assume the quality of the therapy won’t be as good as someone physical.’*

*(Participant 11, completed treatment)*

The assumptions and associated scepticism formed through limited awareness at the start of treatment could lead to anxiety for some participants. This influenced acceptability of telephone delivered treatment, and for some, had an impact on initial willingness to engage. Addressing patient knowledge and understanding at point-of-entry may therefore be fundamental in improving prospective acceptability and behaviour towards telephone-delivered treatment.

### Patient and practitioner skills: effective communication and development of therapeutic alliance are skills-dependent

The ability of both patients and practitioners to communicate effectively over the telephone without visual information or the opportunity for real-time checking of understanding was raised as a significant factor in the effectiveness of sessions, and played an important role in the level of patient acceptance of telephone delivery, specifically around self-efficacy (i.e. belief in own ability to succeed in treatment). Treatment formulation (gaining an understanding of the patients presenting problems) was identified as a significant challenge (TFA: Opportunity Costs, Burden), and could affect session engagement where anxiety around understanding arose (TFA: Affective Attitude). Practitioner misunderstanding of patients’ difficulties reinforced the notion of the superiority of face-to-face treatment, as it was perceived to be less likely to occur face-to-face due to the benefit of visual information. Intrinsically linked to this were challenges in establishing connection. Body language, facial expressions and eye contact were considered important by many in the establishment of a therapeutic bond and effective communication for both parties.

‘*You hear the voice, yeah, but it’s just the interaction like…their body language is I think a really big thing…she was saying I can see how stressful that could have been, but I just felt, oh, okay, you can’t see me though, can you, you know, at times I was literally to tears, whereas if you saw me literally to tears you would act differently towards me, whereas if you’re on the phone you can’t tell if I’m literally to tears. At one point I think I did shed a few tears and she didn’t realise that I was shedding a few tears’.*

*(Participant 10, completed treatment)*

The ability of practitioners to counter such barriers was discussed in terms of specialised training in working by telephone. Active listening and the ability to demonstrate empathy over the telephone (TFA: ethicality) were deemed crucial skills for engaging patients in sessions, establishing good therapeutic relationships and delivering effective treatment.

Disengagement with treatment resulted from practitioner misunderstanding of patient’s problems, and patient perceptions of being offered a ‘one size fits all’ non-personalised treatment. In situations where participants wanted to persevere and drive treatment in a useful direction, it was acknowledged that it was not only practitioners who needed specific skills for telephone treatment. Assertiveness and clear articulation by patients were often felt to be essential on the telephone for sessions to become properly patient-centred (TFA: Self Efficacy), with assertiveness believed an easier feat over the telephone than in person. The potential for more easily achievable patient-directed treatment indicates the potential of telephone delivery to facilitate a power shift to patients.

Handling silence and knowing when to speak and listen were additional telephone-specific issues perceived to contribute to tension (TFA: Opportunity Costs) at the beginning of treatment:

*‘I guess you can’t have that visual feedback, there’s a lot of anticipating. On the phone, it’s much harder to have silences than it is in person, much harder, I find it more anxiety producing on the phone. So I’ll probably be more inclined to just fill it…..you’ve got to be quite skilled in, even just vocalisation, if it’s…you feel like you need to put that in there, just so you know it’s your turn to speak.’*

*(Participant 8, current treatment)*

However, learning how to overcome communication challenges over the course of treatment through increased familiarity with practitioners and session structure led to the development of good therapeutic relationships and shared understanding for most participants, although some reservation remained around whether that would be enhanced in face-to-face treatment (TFA: Perceived Effectiveness). The potential for therapeutic relationships to be stronger face-to-face linked to the importance placed on the role of trust in that development, and the perception that building trust was more difficult in the absence of visual information for some participants. Despite the admission of the development of good relationships over the telephone, bias towards the merits of face-to-face therapy persisted for some:

*‘I think it was a lot harder to build the trust over the phone. Because it was always like that voice in the back of your head, like the person’s got their phone on speaker so then people around them are chatting and laughing. You know that kind of stuff?’ (Participant 11, completed treatment)*

The development of skills through patient-directed self-learning and adaptation to working over the telephone following initial contacts (TFA: Self-efficacy), and trust stemming from practitioner input during sessions (e.g. level of empathy and active listening) appear to be fundamental ingredients in nurturing an effective therapeutic alliance between patient and practitioner. Facilitating patients to drive their own treatment by addressing the important aspects of receiving it over the telephone may improve concurrent acceptability by increasing self-efficacy and thus shift behaviour for more effective engagement.

### Practical and environmental factors: the counterbalance between improved access and practical challenges

Receiving treatment over the telephone was significant in every aspect of participants’ experiences, and data related to the environmental context and resources domain of the TDF mapped to all seven TFA constructs. Effects on communication, the development of therapeutic relationships and thus participants’ engagement were all heavily influenced by telephone as a mode of treatment. Positive implications focussed around accessibility, flexibility and convenience (TFA: Burden, Opportunity Costs), although the idea that telephone treatment could be too accessible was also raised, expressed in terms of intrusiveness, where participants had to immediately return to their usual day (e.g. when taking calls during work time).

In addition to those who opted for telephone for reasons of flexibility and convenience, others with an initial desire for face-to-face were able to articulate the benefits of telephone treatment (i.e. convenience), which had not been initially considered (TFA: Intervention Coherence). Anonymity of telephone treatment - enabling patients to ‘open up’ more easily and avoid stigma associated with visiting a clinic was highlighted as important (TFA: Affective Attitude). A few participants spoke about how the facility to access treatment by telephone had been vital to them due to their cultural context (e.g. where accessing treatment could be frowned upon). Telephone provided privacy and the opportunity to completely conceal treatment (i.e. from family members), which could mean the difference between having and not having treatment.

Practical disadvantages with telephone delivery often centred on physical resources used for ‘homework’, and not patient-practitioner interaction. Receipt of documents containing information about the techniques to practice between sessions was commonly reported as troublesome (i.e. delayed post, undelivered emails). Although some participants reported no issues and found the process acceptable, others spoke about the inconvenience of receiving various different documents over the weeks which were easy to misplace. In contrast, some participants felt overwhelmed by receiving larger workbooks at the beginning of treatment, which could cause confusion, and often contained irrelevant information, reinforcing the lack of personalisation (TFA: Burden).

*‘I think the information he sent me, very often it was pages and pages of all different kinds of mental health problems…. if it was something a bit more specific or relevant to me…email me, send me it, but I don't want to sit there reading hundreds of pages….It was just too much.’*

*(Participant 23, partially completed treatment)*

Elements of session format were also recognised by some as problematic, in particular, completion of outcome measures, which could be perceived as a waste of time (TFA: Opportunity Costs). General acceptance of the necessity for completion (i.e. for tracking recovery for the practitioner) was counterbalanced by a dislike by some for the way in which questions were tackled over the telephone (TFA: Affective Attitude). Interest in ways to expedite or avoid completing measures during sessions was high, with enthusiasm expressed for the opportunity to complete measures independently before a session (as some had experienced during previous face-to-face therapy) to save time and to avoid feeling ‘put on the spot’. A number of participants expressed no issues with completion of outcome measures, however this was described in relation to the usefulness of revisiting scores. Participants discussed how having the ability to track their own scores would be useful for reviewing their progress, and might help keep them on track with their treatment (TFA: Self Efficacy). Although a few participants found their practitioner used the outcome measures to generally reflect on their progress, none of the participants interviewed had been given the opportunity to visually review their previous scores (i.e. graphically), which had been found to be a useful activity in previous face-to-face treatment.

‘*I must admit, every time we did the questions, I, kind of, wished that I had remembered what it was what I’d said the previous week in the session and [practitioner name] gave no indication, maybe a couple of times she might have mentioned things if things had changed perhaps not in the right direction… she’d, sort of side step from the question and just ask a little bit more….But, yeah, just on a personal note, I would probably have responded quite well to knowing how I was progressing from that.’*

*(Participant 14, completed treatment)*

Completion of outcome measures was one feature that contributed to the perception of treatment on the telephone feeling rigid, business-like and scripted. Notions that practitioners were *‘reading from a script’* gave the impression of generic treatment, further exacerbated by strict adherence to session length, which had a downstream effect on self-efficacy in treatment (TFA: Ethicality, Self-Efficacy, Perceived Effectiveness). One participant spoke about how he believed it might be a lack of training which was responsible for a rigid impression.

*‘it still is routine, like now we're going to go to this page and we're going to do this and you're going to this, and if it didn't necessarily work I felt that sometimes they didn't know what to say necessarily, do you know what I mean? …I think they did everything they're kind of trained to do, everything they knew how to do.’*

*(Participant 2, completed treatment)*

Participants who did not perceive their treatment to have been inflexible described the skill of their practitioner in allowing sessions to be shaped around the patient while still completing the required components of the appointment.

‘*[each]* s*ession definitely built on the previous one, so we’d have a bit of time to talk through whether I’d managed any of the techniques or anything like that, but I didn’t feel as though the sessions were mapped out in advance, you know, I don’t feel as though [practitioner name] had any plans to, kind of, steer me in any particular direction… I felt as though she had this bank of experience and knowledge and techniques that she could draw upon to assist me’*

*(Participant 14, completed treatment)*

Targeting the specific behaviour of those practitioners presenting a rigid or scripted impression could therefore alleviate the sense of inflexibility of treatment to influence patient acceptability. Similarly, tackling the practical aspects of between-session work for patients could also facilitate a shift in behaviour through increased concurrent acceptability of telephone-delivered treatment.

### Beliefs: overcoming preconceptions and inaccurate beliefs through experience

Most notable was the contrast in initial expectations and reservations of some participants, with reflections on experience. Descriptions of ‘bumpy starts’ and low expectations for telephone treatment improved during the course of treatment (TFA: Perceived Effectiveness, Intervention Coherence, Affective Attitude), illustrating a contrast between initial low prospective acceptability before treatment and reasonably high retrospective acceptability after completion. Apprehension about practitioner ability, establishing relationships, communicating and reaching a shared understanding, for many, was dispelled which enabled effective engagement in sessions and feelings of having benefited from treatment (TFA: Perceived Effectiveness, Ethicality, Self-efficacy).

*‘My expectations were surpassed through the process. And as I said before, I wasn’t a fan of it before because I’d never heard of it. And the concept of it frightened me and it didn’t really make sense in my head because I had never heard of it. Before I was just like, okay, cool, face-to-face therapy, that’s what it always is. Then at the end of it, I was very much changed by it.*

*(Participant 11, completed treatment)*

Changes in patient perspective of telephone treatment were in part attributed to individual practitioner merits, expressed in terms of seeming genuine, and being skilled and supportive, which helped people to relax and invest in their treatment. Equally, those who did not report such positive experiences often attributed blame to the practitioner and not the mode of treatment, citing issues such as compatibility and practitioner demeanour.

Importantly, telephone was, by most, considered an appropriate way to deliver treatment, due to the format of treatment provided (TFA: Intervention Coherence, Perceived Effectiveness). Initial lack of understanding about what treatment would entail contributed to the belief that it would be ineffective, however realisation of the guided self-help nature and structure of sessions shifted opinion:

*‘I don’t think you could easily do [counselling] over the phone…it’s where something’s very personal and you need to get… you want to feel… through body language and that kind of thing….So I think because it was learning tools and techniques rather than going into a load of detail about what your problems were…you know... So I think that’s why it worked because it was a lot of self-learning, you know. So I think because it was teaching you how to take on these skills, that was what made it perfectly acceptable to have it over the phone.’*

*(Participant 18, completed treatment)*

Despite numerous positive turnarounds, there remained some who felt telephone treatment could not replace face-to-face, although most descriptions of this were in terms of people with more complex mental health issues (e.g. trauma) (TFA: Perceived Effectiveness, Affective Attitude, Ethicality). Numerous participants agreed they would return to telephone treatment in the future if necessary, and would recommend it to others; however, some maintained they would want face-to-face treatment if required in the future, even when they had considered treatment effective. The only tangible reasons participants were able to offer for this centred on a pervasive idea that *‘it’s just not as personal’* as face-to-face treatment or a general dislike of communicating by telephone (TFA: Opportunity cost). Such reflections indicate the importance of addressing patients’ beliefs and perceptions of both treatment and practitioners for improved acceptability of and engagement with telephone treatment.

## Discussion

Best practice in intervention development is underpinned by strong evidence and appropriate theory [29]. This study is the first to explore patient views of telephone-delivered treatment in IAPT services from a dual acceptability and behaviour change theory perspective.

Our findings have highlighted that although an array of opinions and experiences of telephone treatment are reported, there is a distinct group that describes similar experiences. Preliminary reluctance to engage in telephone treatment stemming from lack of knowledge and understanding can be exacerbated by the impression of being ‘sold’ an inferior treatment, or feelings of having no choice but to opt for telephone to avoid long waiting lists for face-to-face treatment. Once treatment has commenced, initial difficulties engaging in sessions due to lack of confidence in treatment efficacy, the absence of additional information afforded by face-to-face treatment, and the belief a therapeutic connection will be difficult to establish over the telephone, contributes to the low level of acceptability felt by some. However, as treatment progresses, there appears a general shift in attitude by this group. Realisation that treatment over the telephone can be effective, and the development of a better understanding of treatment begins to diminish the importance placed on seeing a practitioner face-to-face. Although there were individuals for whom general acceptability did not increase, this was attributed to issues with compatibility with practitioners, or enduring dislike for telephone communication in general. Where participants reflected positively on their experiences, the benefits of flexibility were expressed differentially across age groups, where older participants highlighted the value of being able to remain at home and avoid travel, while younger participants articulated the advantages of fitting treatment around work and childcare.

The use of the telephone for the delivery of psychological interventions has undoubtedly enabled wider access to treatment for patients [10–12]. The evidence for clinical effectiveness and acceptability of the telephone as a mode of treatment has been established [13, 30]; challenges, however, remain around uptake and engagement. In order to gain a better understanding of the reasons, this study has explored patient perceptions of telephone treatment and identified a number of areas important to target to increase acceptability. The relationship between acceptability and behaviour change is interdependent; in order to change behaviour something must be acceptable, and in turn, change in behaviour can cause something to become more acceptable.

The influence of knowledge on patient attitudes and behaviour towards treatment compliance and attendance has been well documented in a variety of long term conditions including diabetes [31], kidney disease [32], heart disease [33] and cystic fibrosis [34]. Our data have similarly shown that addressing patient knowledge and understanding of treatment could be the key to initiate change. Inadequate awareness of treatment, based on assumptions derived from erroneous information, results in poor prospective acceptability of treatment. Targeting deficits in knowledge to facilitate accurately informed entry into treatment is important in order to positively engage patients from the outset. Promoting the merits of telephone treatment and demonstrating practitioner experience and confidence could emphasise the validity of telephone delivery, which can increase acceptability by inciting automatic trust [11].

Practical considerations - perceived in some terms as the most simple to address - may significantly improve concurrent acceptability (during) treatment. Previous work in chronic pain has demonstrated improved engagement with and completion of pain diaries when completed electronically [35]. Changing therapeutic resources to facilitate use in telephone sessions, and exploring approaches for more acceptable ways to complete routine outcome measures must be a focus in any behaviour change intervention for improving telephone treatment in IAPT.

Patients’ confidence in their ability to engage effectively in telephone treatment is an additional key target for changing behaviour. Perceived difficulties in communication and understanding, and absence of non-verbal communication and visual information limit the level of acceptability of telephone treatment due to perceived burdens and opportunity cost. Empowering patients to drive treatment by making sessions less practitioner led, more flexible and personalised, and redressing the balance between telephone delivered and face-to-face treatment (i.e. by providing the option to receive information about the practitioner, such as a photograph) could also support the development of a therapeutic relationship by facilitating familiarity and reducing the perceived barriers of telephone treatment.

Although positive reflections summarise the experience of telephone treatment by most, many nevertheless retain the preference of face-to-face for hypothetical future treatment. Understanding why face-to-face remains a preferred option, despite positive experiences over the telephone, is crucial. Ingrained perceptions of desired treatment are reinforced by referrers’ (i.e. GPs) explanation of the treatment, and exacerbated by the way treatment is sold to patients who often opt in essentially as they see it as better than nothing when faced with the prospect of a lengthy wait for face-to-face treatment. Promoting telephone treatment by its shorter waiting lists may perpetuate the belief that it is unpopular and inferior. Presenting it instead as legitimate routine provision might lead to greater trust and acceptability, which in turn may improve patient engagement in telephone treatment.

### Strengths and limitations

Utilising two theoretical models to explore the data was a strength of this study. Considering data across the two frameworks has provided greater specificity with regard to the areas important to target to improve patient acceptance for increased engagement in treatment. The TFA provided a structured theory around which to explore patient acceptability of specific elements of telephone-delivered treatment, without presuming acceptability based on patients’ behaviour [23]. The TDF facilitated in mapping the study findings into coherent areas to target for change providing a theoretically robust premise for later-stage implementation studies [24, 25].

Another strength of this study was the research-practitioner collaboration between KR (an experienced mental health qualitative researcher) and KA (a PWP and research assistant). Integrating this skill set provided two perspectives that cultivated a more comprehensive understanding of the data. Disseminating and implementing research into health care practice can be challenging [36], and thus practitioner involvement in this study design will help bridge the gap between research and practice [37].

This study was limited by the retrospective accounts from patients, as receipt of treatment could have been up to 12 months pre-interview, and because over 75% of participants were interviewed by telephone, indicating a possible bias towards telephone communication. In addition, our sample was predominantly White British, and the views of patients who had chosen not to wait for telephone treatment were not sought. Future work may strengthen findings by studying telephone session interactions in more detail i.e. via conversation analysis [38], and by purposively selecting patients from a wider range of ethnic backgrounds, and those who failed to commence treatment following assessment.

## Conclusion

Addressing patient knowledge and understanding is key to initiate a cycle of change to improve the acceptability and thus uptake and sustained engagement with telephone treatment. Improving practical aspects of treatment, along with targeting key practitioner and patient skills can facilitate better engagement and improve patients’ trust in treatment efficacy. Ultimately, increasing patient acceptability of telephone treatment by improving the patient experience will contribute to better quality telephone –delivered psychological treatment.

## Supplementary information

**Additional file 1.** Patient data codes mapped to TDF domains and TFA constructs.

## Data Availability

The dataset generated and analysed during the current study are not publicly available due to potential for breach of anonymity, but are available from the corresponding author on reasonable request.

## Abbreviations

EQUITy: Enhancing the quality of psychological interventions delivered by telephone
GP: General Practitioner
IAPT: Increasing access to psychological therapies
TDF: Theoretical domains framework
TFA: Theoretical framework of acceptability
UK: United Kingdom
